# Supplementary material for: Isolation and Identification of Pentalenolactone Analogs from Streptomyces sp. NRRL S-4
Source: Molecules. 2021 Dec 5;26(23):7377. doi: 10.3390/molecules26237377 (PMC8659275; doi:10.3390/molecules26237377)

Supplementary Materials

Isolation and Identification of Pentalenolactone Analogs from *Streptomyces* sp. NRRL S-4

Huanhuan Li[1,2,†], Hongji Li[1,†], Shuo Chen[1,2], Wenhui Wu[2,*], and Peng Sun[1,*]

[1] Department of Phytochemistry, School of Pharmacy, Second Military Medical University, 325 Guo-He Road, Shanghai 200433, People's Republic of China;

[2] Department of Marine Bio-Pharmacology, College of Food Science and Technology, Shanghai Ocean University, 999 Huchenghuan Road, Shanghai 201306, People's Republic of China

* Correspondence: sunpeng78@126.com (P.S.); whwu@shou.edu.cn (W.W.); Tel.: +86-21-81871259 (P.S.)

† These authors contributed equally to this work

Table of Content

Table S1. Biosynthetic gene clusters in *Streptomyces* sp. NRRL S-4 3

Figure S1. The optimized conformers of 1 and Boltzmann populations (>1%)........ 4

Figure S2. The optimized conformers of 2 and Boltzmann populations (>1%)........ 5

Figure S3. MS and NMR spectra of 1 6

Figure S4. MS and NMR spectra of 2 11

Table S1. Biosynthetic gene clusters in *Streptomyces* sp. NRRL S-4

Cluster No.	NP type	Size (kb)	Most similar known cluster	similarity
Cluster 1	terpene	25.5	Isorenieratene	100
Cluster 2	NRPS	50.0	Mirubactin	57
Cluster 3	bacteriocin	111.5	Oxazolomycin	18
Cluster 4	T1PKS	94.9	cremimycin	30
Cluster 5	terpene	26.6	Hopene	69
Cluster 6	T1PKS	205.0	Rifamycin	21
Cluster 7	bacteriocin	11.3	-	-
Cluster 8	NRPS	132.6	Xiamycin A	72
Cluster 9	butyrolactone	65.5	Chlorizidine A	11
Cluster 10	siderophore	14.6	ficellomycin	3
Cluster 11	terpene	21.1	-	-
Cluster 12	bacteriocin	10.2	-	-
Cluster 13	PKS/NRPS hybrid	50.7	BD-12	17
Cluster 14	butyrolactone	10.9	Zorbamycin	6
Cluster 15	PKS	41.0	rustmicin	33
Cluster 16	lantipeptide	23.1	Thioviridamide S-4	100
Cluster 17	terpene	21.0	Pentalenolactone	58
Cluster 18	terpene	21.0	-	-
Cluster 19	ectoine	10.4	Ectoine	100
Cluster 20	terpene	21.1	Steffimycin	16
Cluster 21	PKS/NRPS hybrid	52.2	Cinnamycin	14
Cluster 22	ectoine	10.4	Ectoine	100
Cluster 23	PKS/NRPS hybrid	109.5	RP-1776	6
Cluster 24	melanin	10.5	Melanin	100
Cluster 25	T2PKS	49.2	Spore_pigment	83

Figure S1. The optimized conformers of **1** and Boltzmann populations (>1%).

	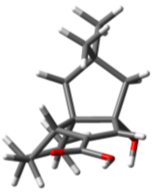	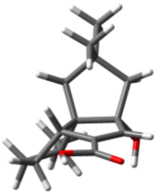	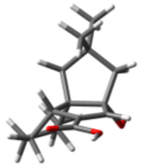
Conformer	I	II	III
Boltzmann population (%)	25.36	11.40	2.29

	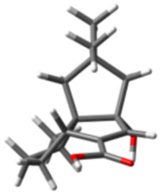	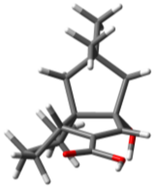	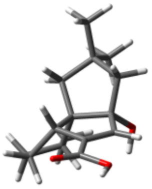
Conformer	IV	V	VI
Boltzmann population (%)	11.44	25.44	4.35

	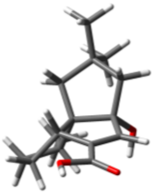	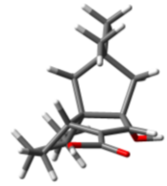	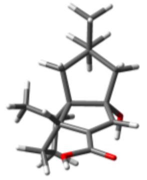
Conformer	VII	VIII	IX
Boltzmann population (%)	1.79	1.38	3.05

	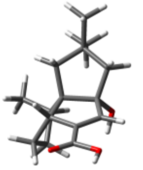	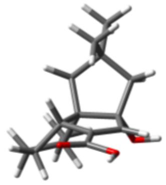	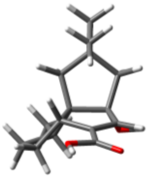
Conformer	X	XI	XII
Boltzmann population (%)	7.10	2.48	1.36

	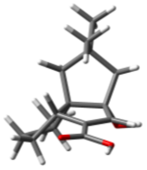
Conformer	XIII
Boltzmann population (%)	2.57

Figure S2. The optimized conformers of **2** and Boltzmann populations (>1%).

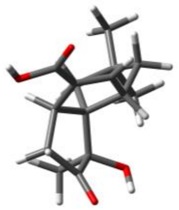 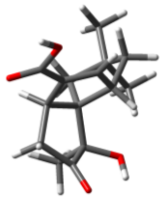 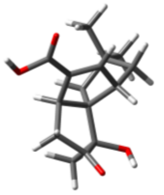

Conformer	Ⅰ	Ⅱ	Ⅲ
Boltzmann population (%)	15.50	57.34	5.87

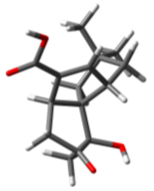

Conformer	Ⅳ
Boltzmann population (%)	21.29

Figure S3. MS and NMR spectra of **1**

a) IR spectrum

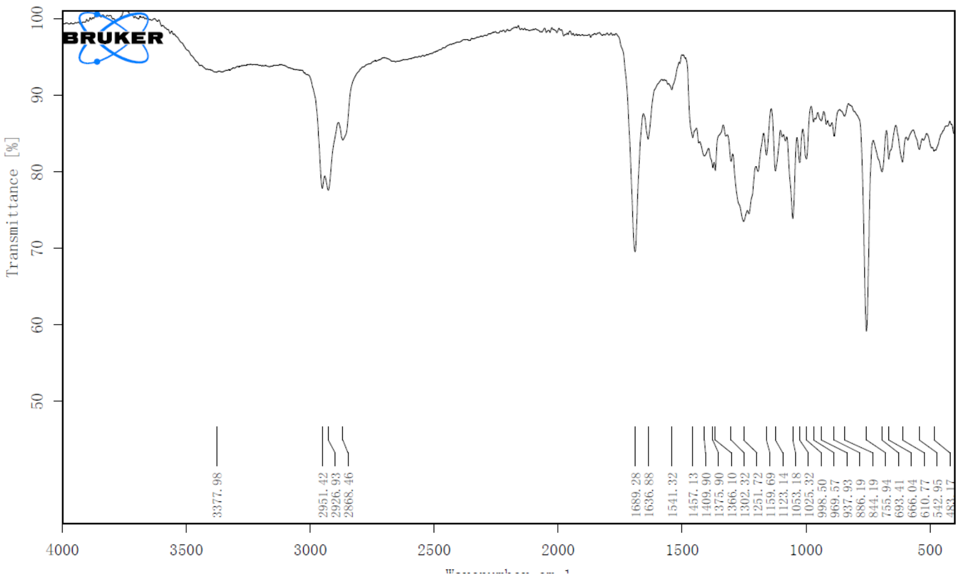

b) HRESIMS spectrum

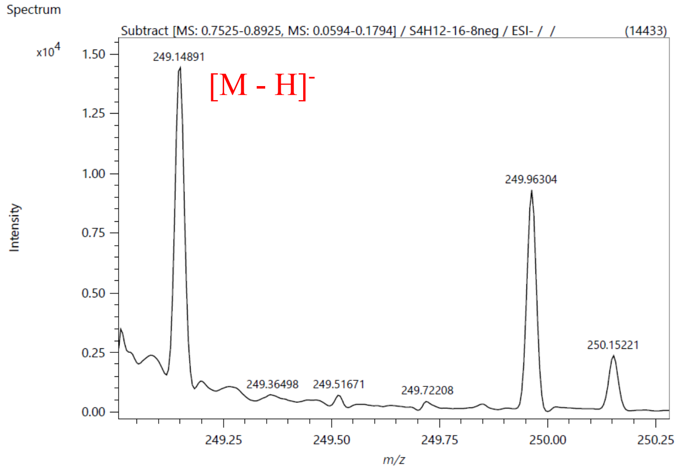

Results

Mass	Intensity	Intensity [%]	Formula	Calculated Mass	Mass Difference [mDa]	Mass Difference [ppm]	DBE
249.14891	14432.76	6.94	C15 H21 O3	249.14852	0.39	1.58	5.5

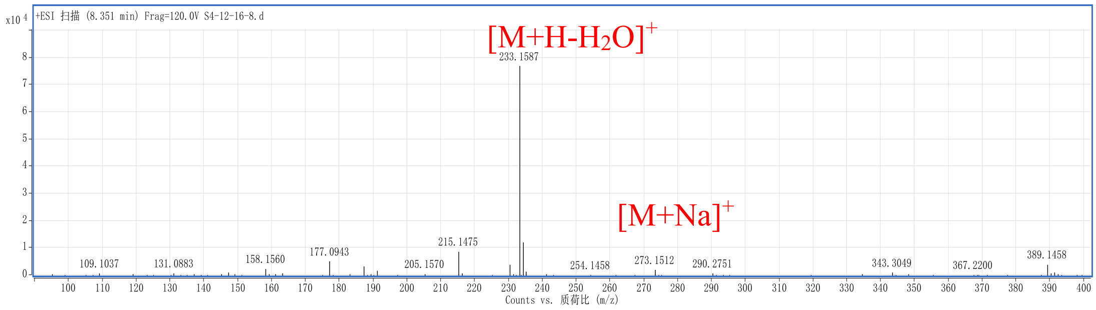

c) ^{1}H spectrum

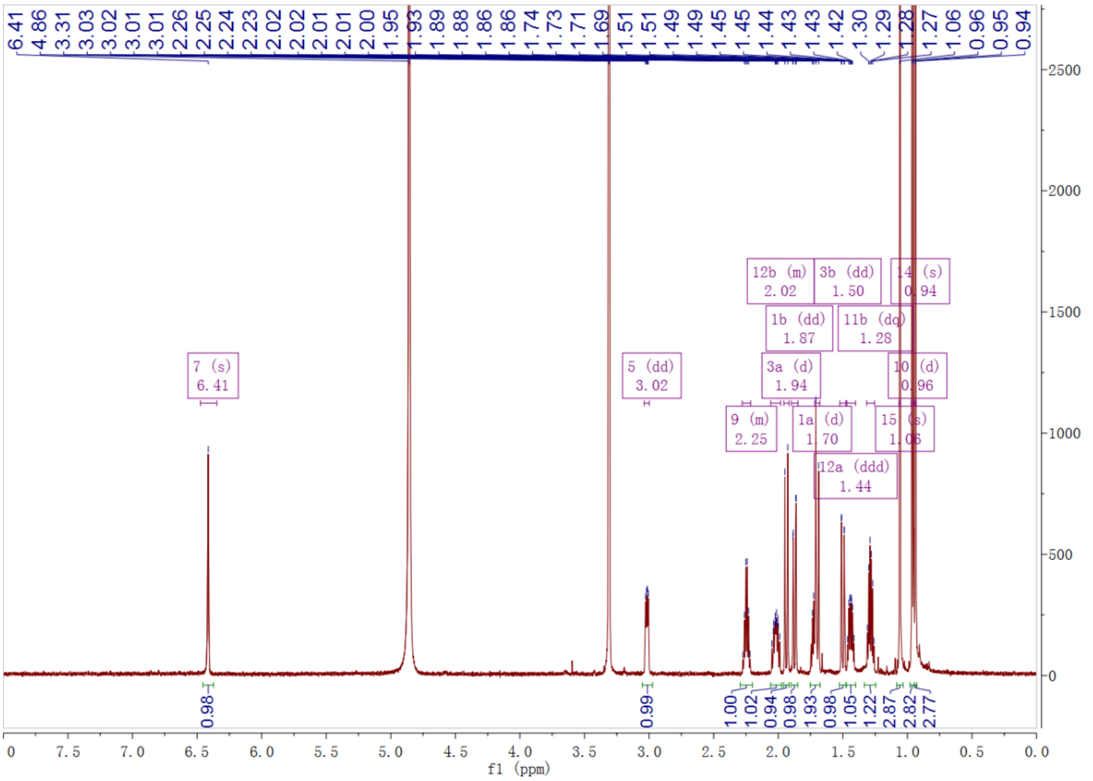

d) ^{13}C spectrum

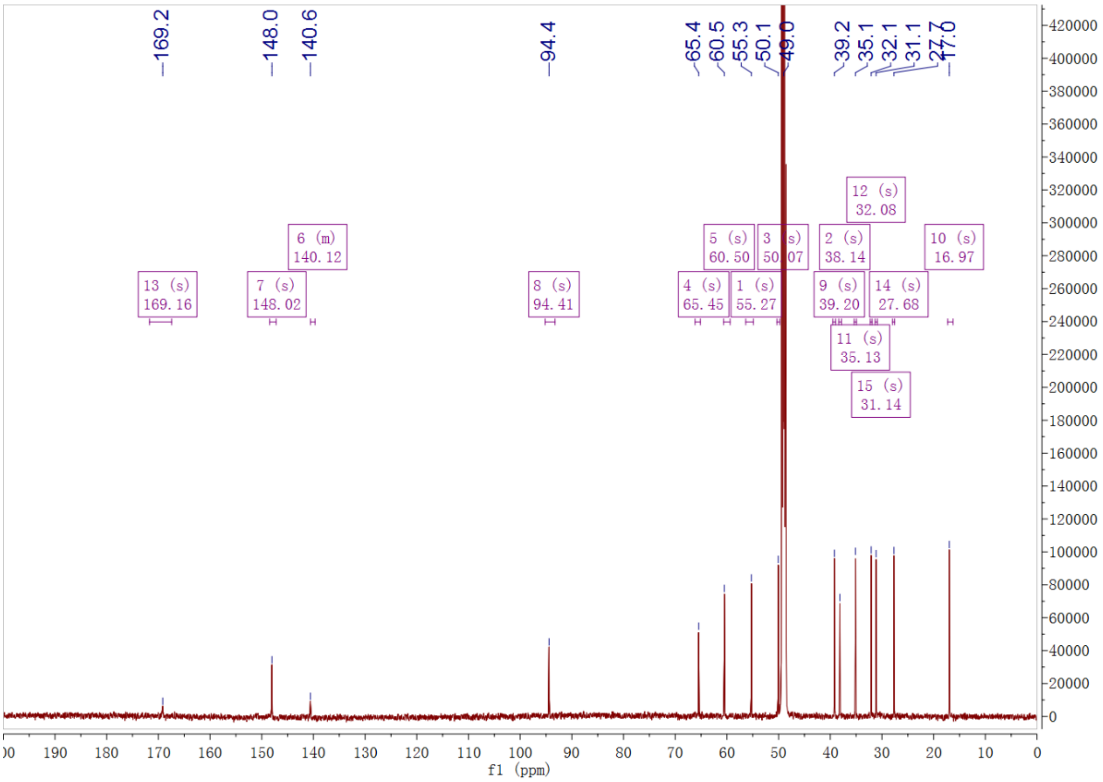

e) COSY spectrum

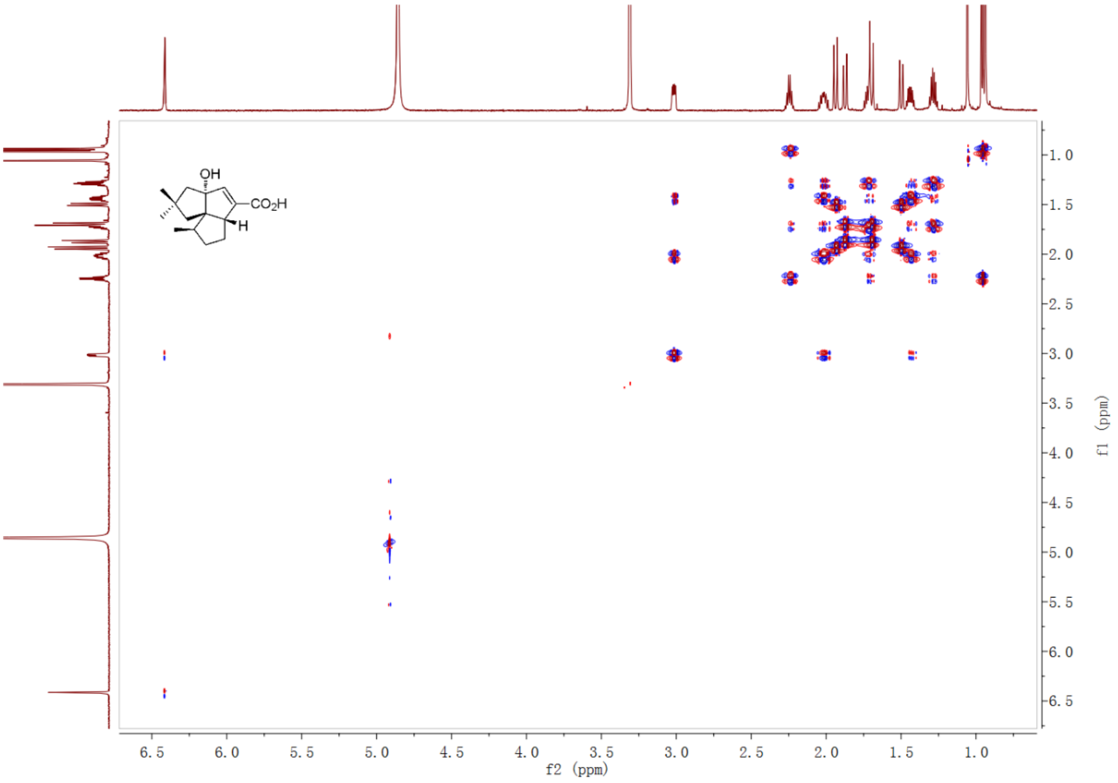

f) HMQC spectrum

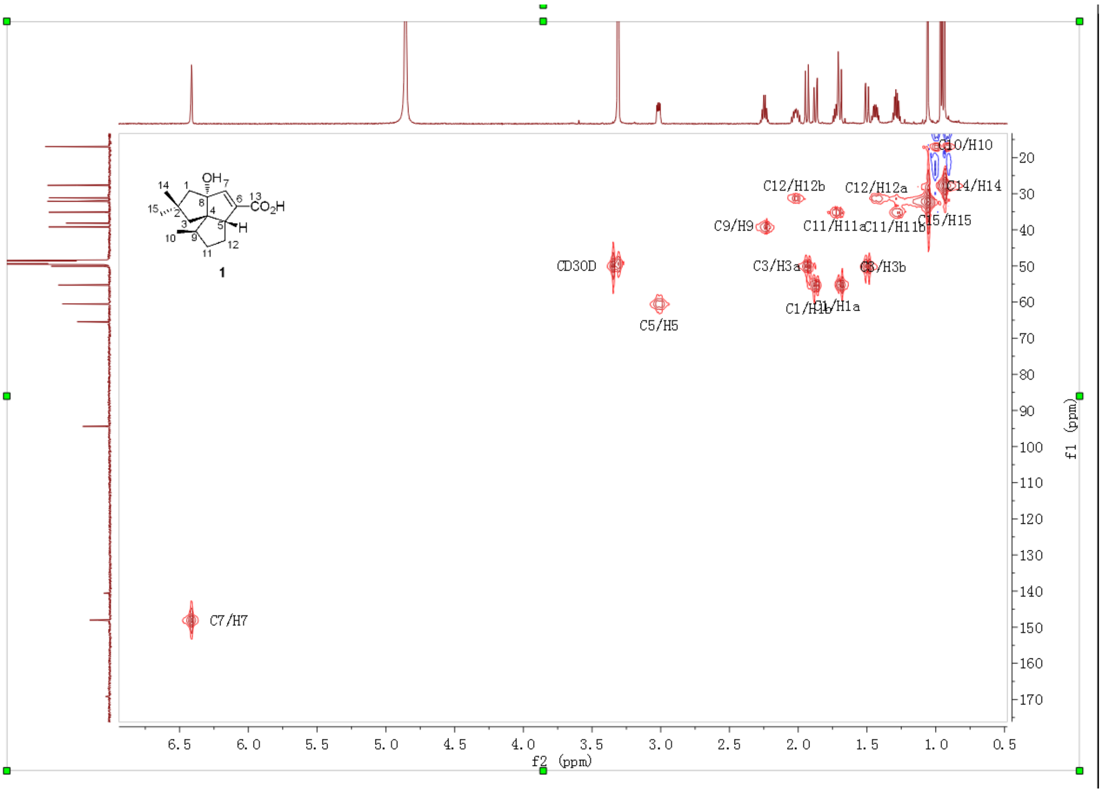

Enlarged HMQC spectrum for CH_2 correlations

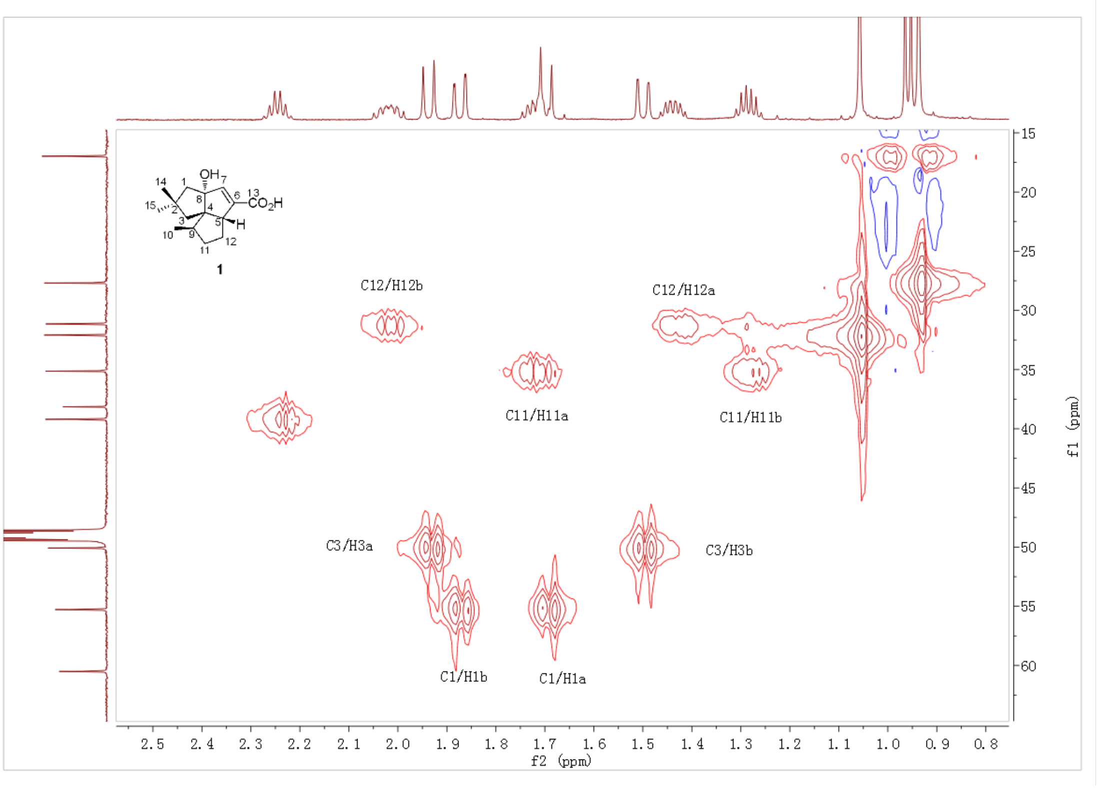

g) HMBC spectrum

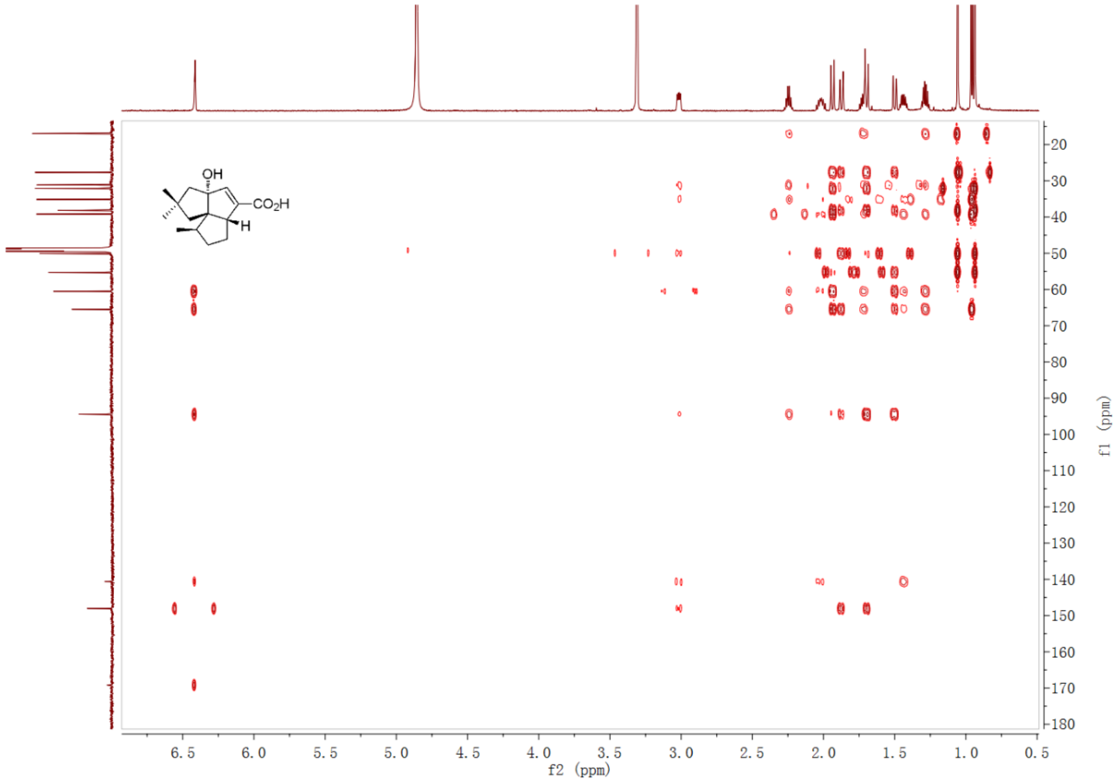

h) NOESY spectrum

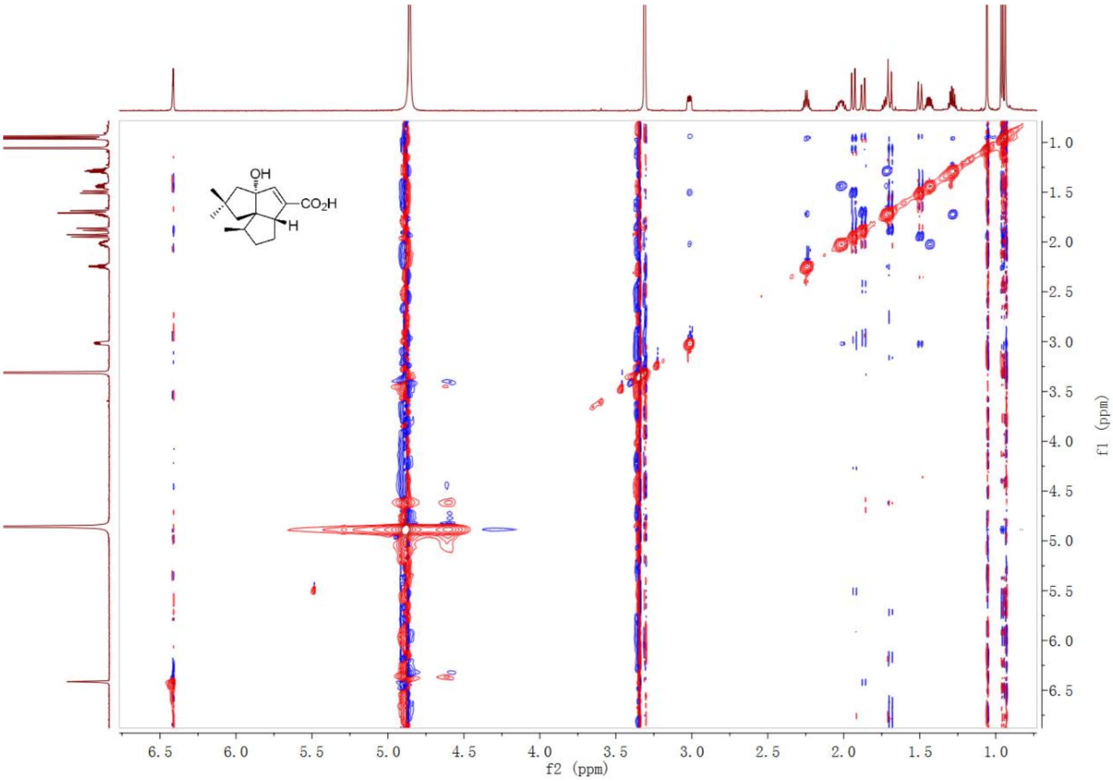

Figure S4. MS and NMR spectra of **2**

a) IR spectrum

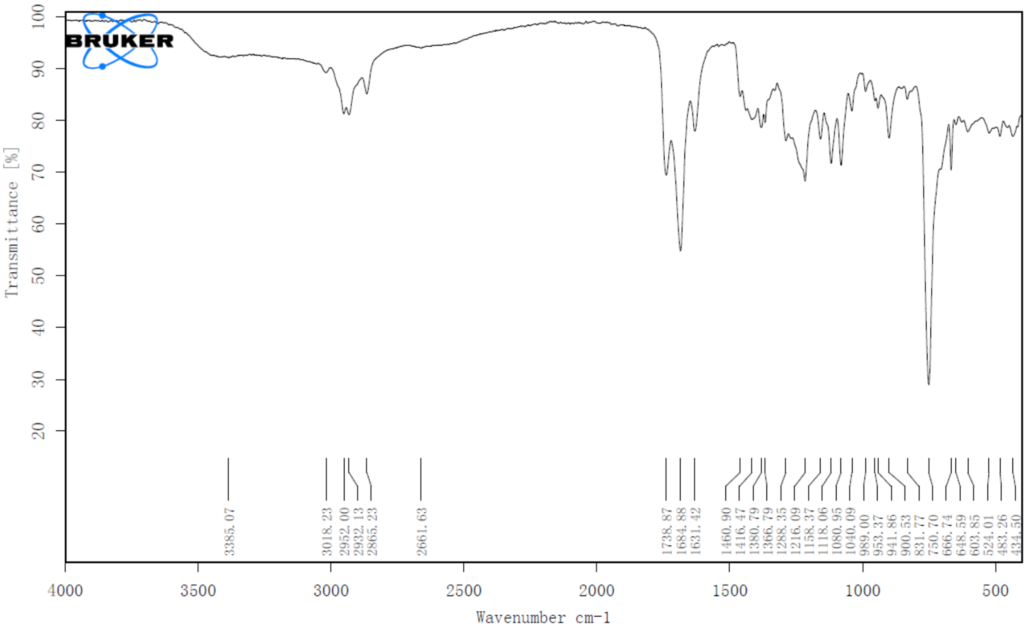

b) HRESIMS spectrum

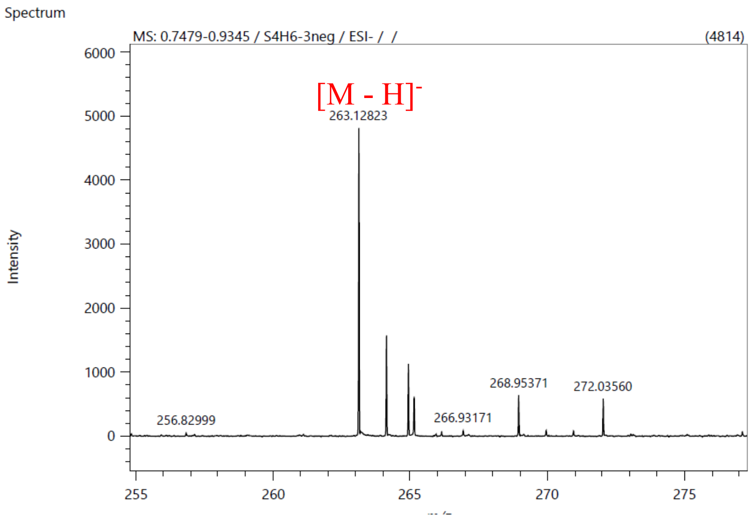

Results

Mass	Intensity	Intensity [%]	Formula	Calculated Mass	Mass Difference [mDa]	Mass Difference [ppm]	DBE
263.12823	4813.93	4.69	C15 H19 O4	263.12779	0.44	1.68	6.5

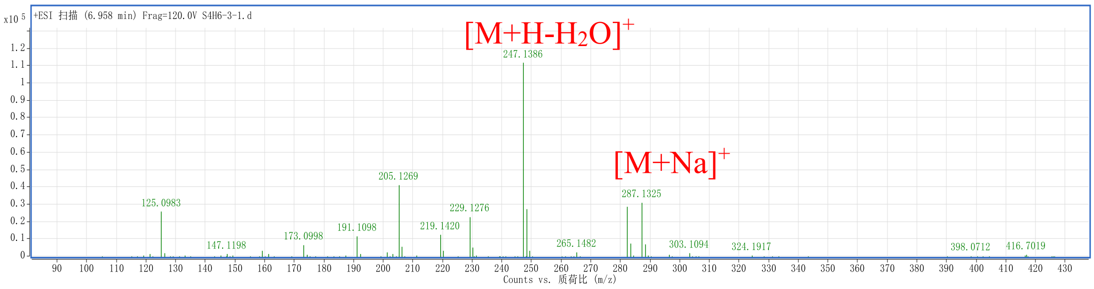

c) ^{1}H spectrum

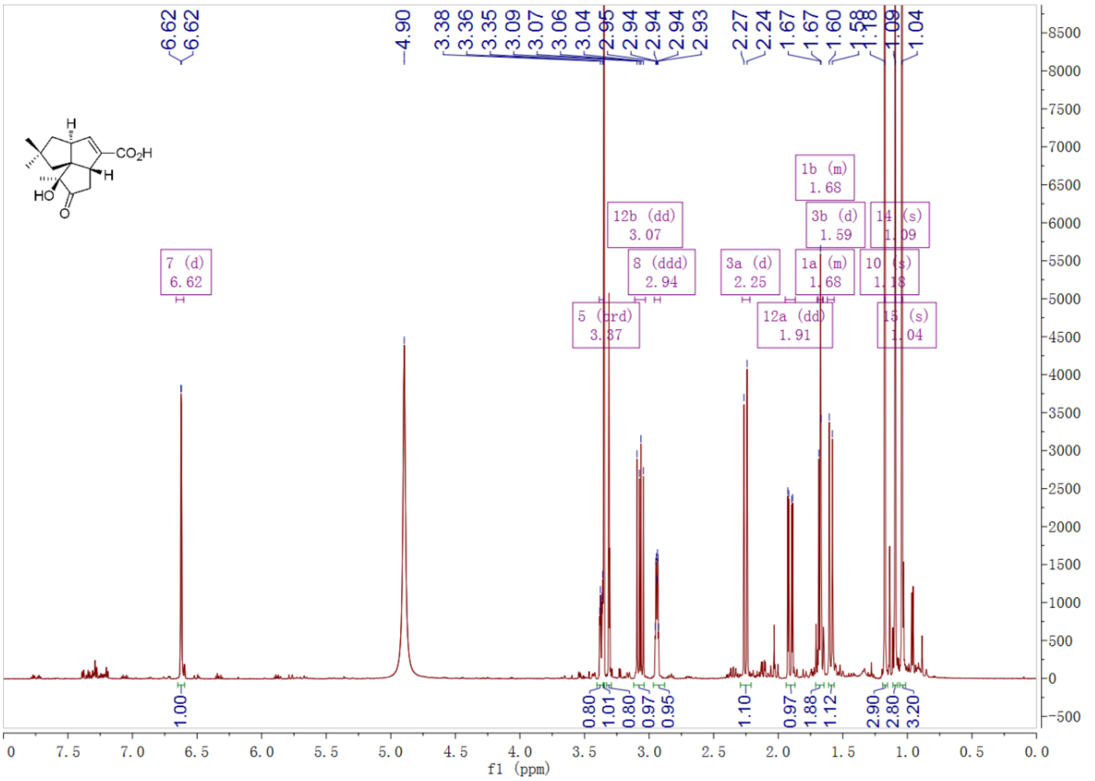

d) ^{13}C spectrum

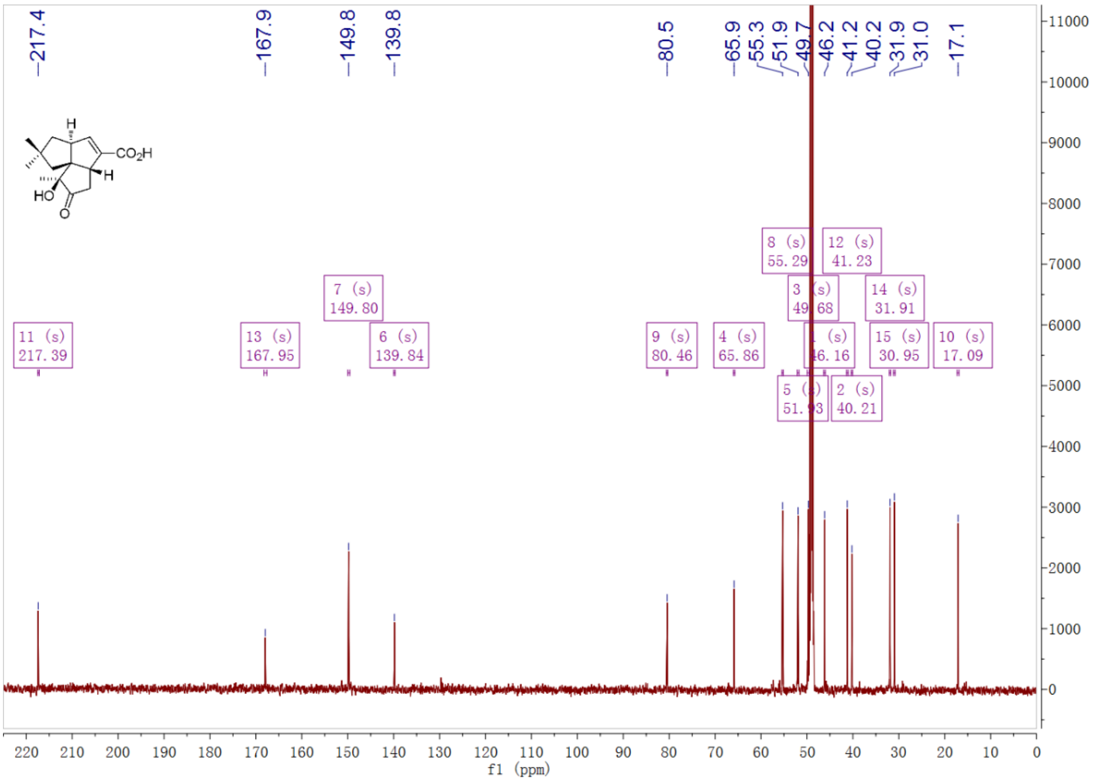

e) COSY spectrum

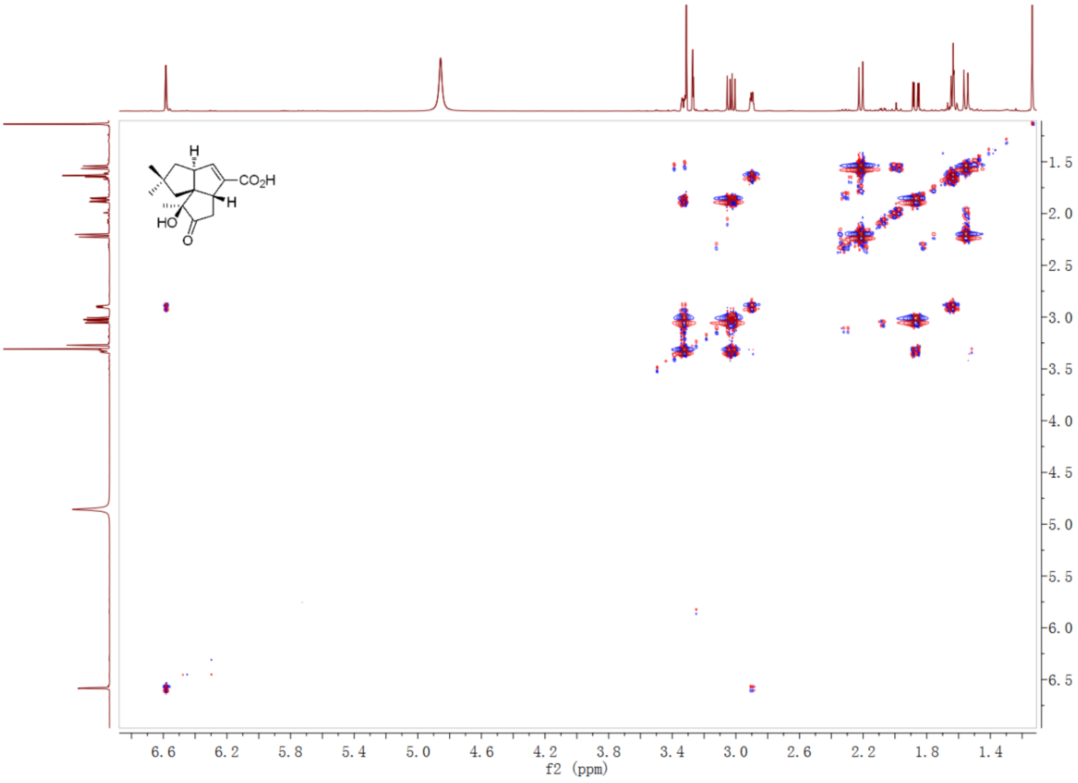

HMQC spectrum

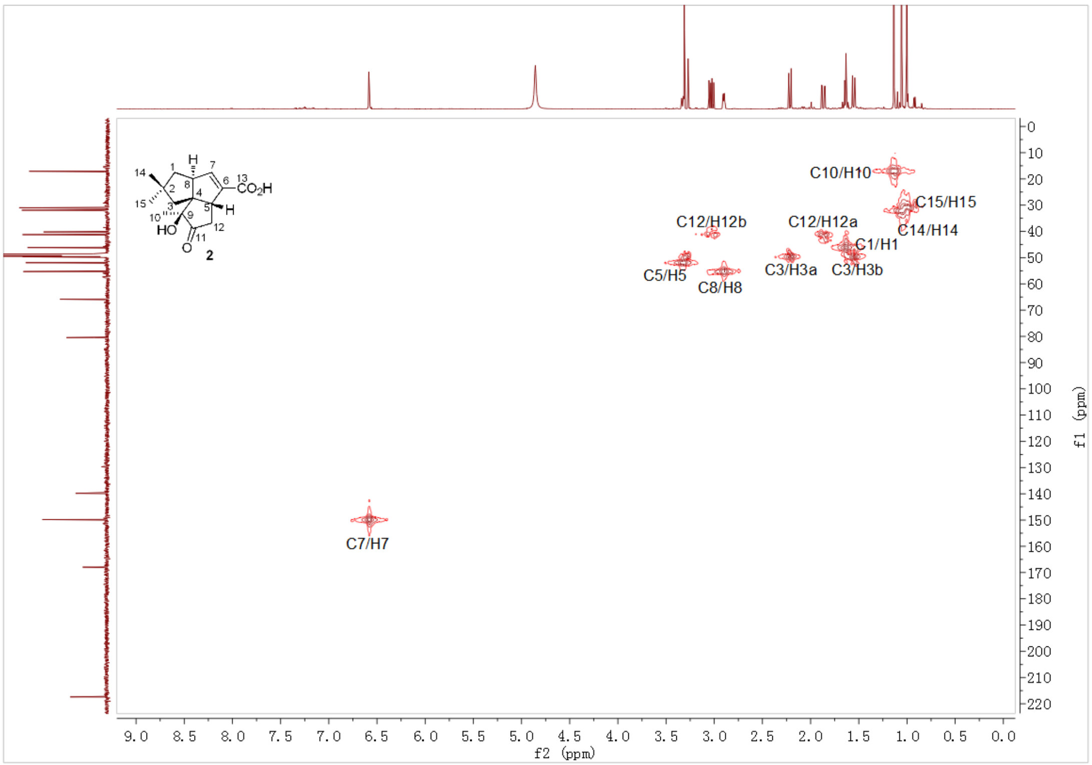

Enlarged HMQC spectrum for CH_2 correlations

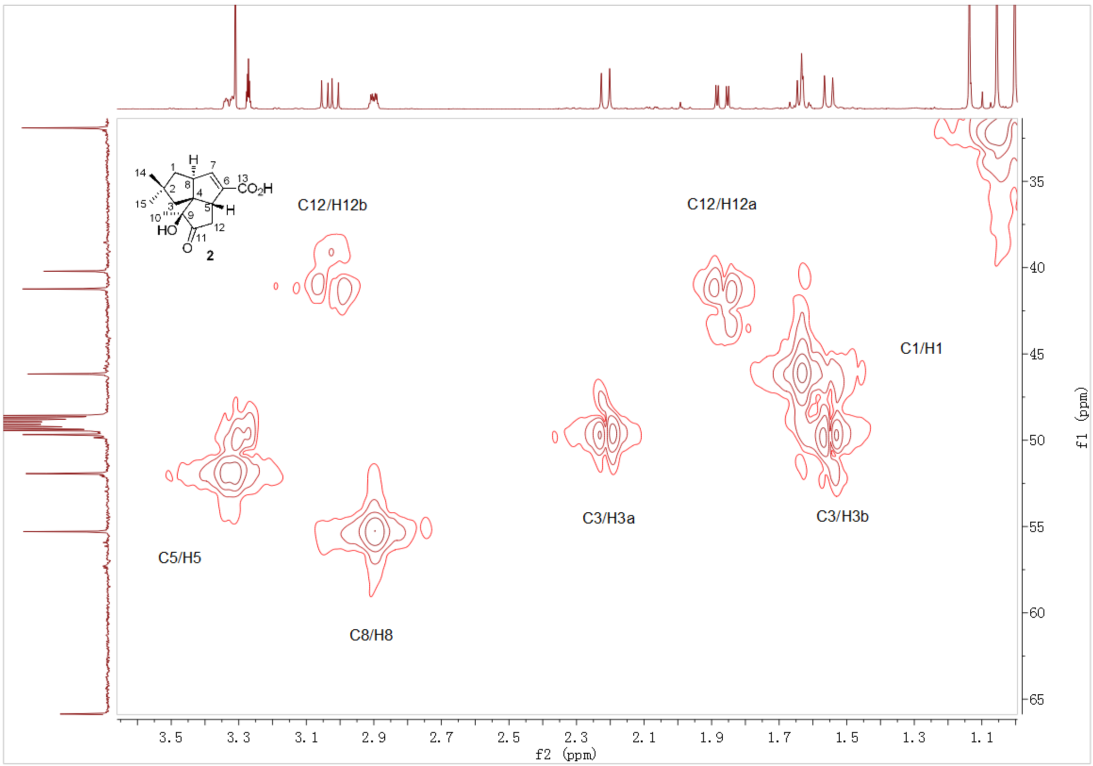

f) HMBC spectrum

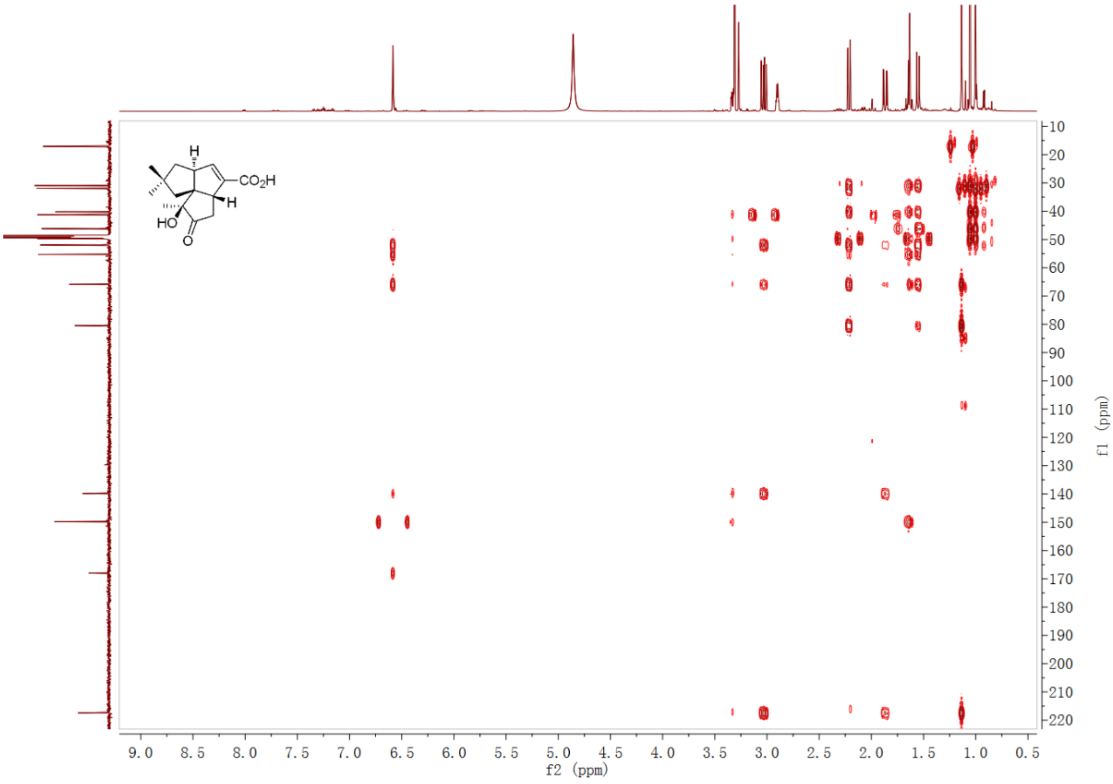

NOESY spectrum

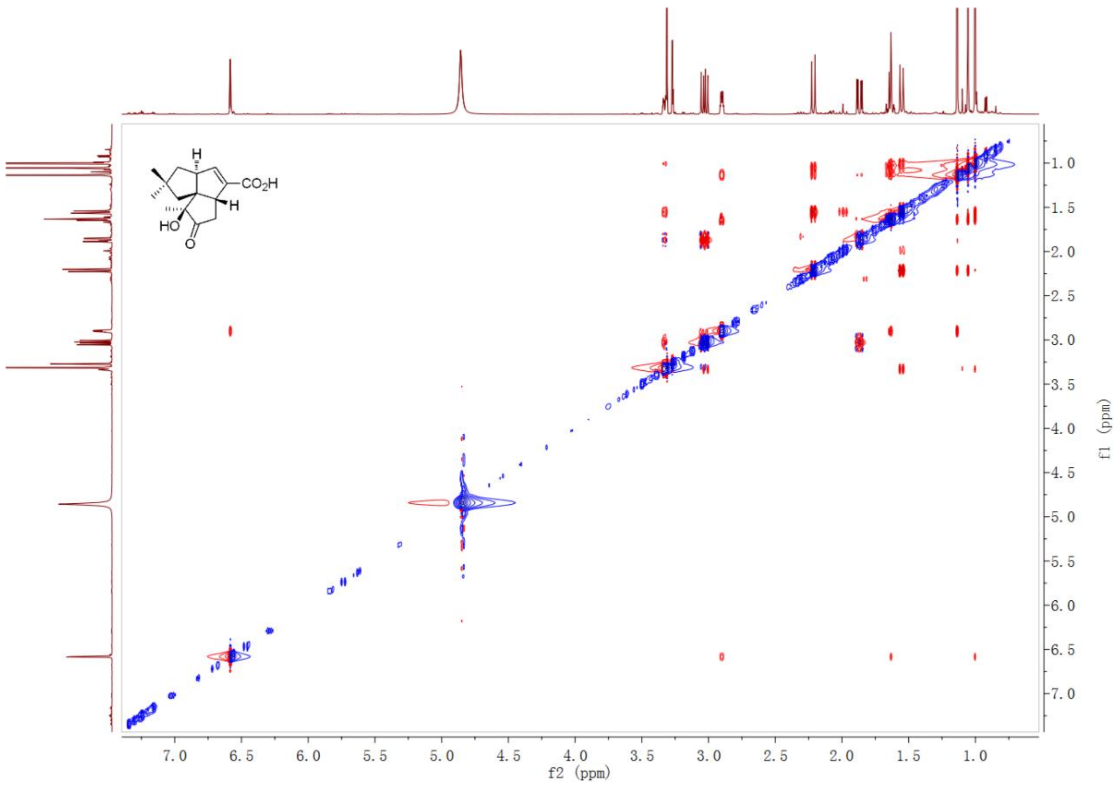

Supplement: Supplementary file 1 [file molecules-26-07377-s001.zip › molecules-1461968-supplementary.pdf]
